# Recent Developments in Photoluminescent, Photothermal and Photocatalytic Nanomaterials

**DOI:** 10.3390/nano13121888

**Published:** 2023-06-19

**Authors:** Zhixing Gan

**Affiliations:** Center for Future Optoelectronic Functional Materials, School of Computer and Electronic Information/School of Artificial Intelligence, Nanjing Normal University, Nanjing 210023, China; zxgan@njnu.edu.cn

Photoactive nanomaterials exhibit myriad customized properties, including a photon converting ability, specific surface area, physicochemical stability, and chemical reactivity, making them appealing for a wide range of practical applications. Photoactive nanomaterials can convert photon to photon (photoluminescence) [1], heat (photothermal effect) [2], and separated charged carriers (photocatalytic reaction, photovoltaic effect) [3], which is very important for the manipulation of light and the utilization of solar energy. This Special Issue collates eleven papers, including nine research articles and two review articles, on recent research progress in emerging photoluminescent (PL), photothermal, and photocatalytic nanomaterials, and their applications in optoelectronics and energy conversion. 

The photoluminescent materials investigated include crystalline tris(8-hydroxyquinoline) aluminum (Alq3) micro-rods (MRs), Fe^2+^-doped CsPb(Cl_x_Br_1−x_)_3_ nanocrystals (NCs), organosilica films, and Tm^3+^-doped SiO_2_–HfO_2_ [4,5,6,7]. Kim et al. fabricated hybrid Alq3/Ag structures using a self-assembly method under a mixed solution of protic and aprotic polar solvents [4]. An evident PL enhancement of approximately 26 times was obtained due to the localized surface plasmon resonance (LSPR) effects between crystalline Alq3 MRs and Ag nanowires. Rasadujjaman et al. revealed that the PL of organosilica films mainly originates from the carbon-containing components rather than the oxygen-deficient centers [5]. They showed that the PL intensity could be improved by increasing the porosity and internal surface area. Wu et al. prepared Fe^2+^-doped CsPb(Cl_x_Br_1−x_)_3_ NCs using a hot injection method [6]. Since the defect density was reduced by the Fe^2+^ doping, the full width at half maximums declined, while PL quantum yields (QYs) and photostability increased. Zulfikri et al. deposited Tm^3+^-doped SiO_2_–HfO_2_ in the form of nanofibers (NFs) and thin films (TFs) on a single substrate using the electrospinning and dip-coating methods, respectively [7]. Compared with single-layered NF and TF structures, the composites showed an about tenfold improvement in PL intensity. These articles reported the PL enhancement of different nanomaterials via various approaches, which will not only promote the applications of these PL materials, but also inspire the development of the PL modulation method. 

Two review articles presented content related to photothermal nanomaterials [8,9]. Thermochromic smart windows that can actively regulate solar radiation according to the ambient temperature have important application prospects in the field of lower energy consumption buildings. Both photothermal effects and electrothermal effects can induce the thermochromic process. Thus, a large number of pioneering investigations have been conducted to change the optical performance of smart windows based on photothermal or electrothermal effects at room temperature. Recent advances in photo- and electro-driven thermochromic smart windows are summarized in these two review articles. 

Another main contribution comes in the field of photocatalytic nanomaterials. Meng et al. fabricated black 3D-TiO_2_ nanotube arrays on Ti meshes using a facile electrochemical reduction method [10]. The black 3D-TiO_2_ nanotube arrays attained a maximal photocurrent density of 1.6 mA/cm^2^ at 0.22 V vs. Ag/AgCl with a Faradic efficiency of 100%, resulting in an enhanced photoelectrochemical water-splitting performance. Guan et al. prepared molybdenum-doped thin ZnIn_2_S_4_-containing S vacancies (Mo-doped Sv-ZnIn_2_S_4_) via a one-pot solvothermal method [11]. The cooperation of Mo doping and S vacancies enhanced light absorption and facilitated the separation of the electron-hole, while also providing active sites. Thus, the Mo-doped Sv-ZnIn_2_S_4_ exhibited a high hydrogen evolution rate of 5739 μmol g^−1^ h^−1^ with a high apparent quantum yield of 21.24% at 420 nm. Boaretti et al. explored the effects of combinations of different advanced oxidation processes on the degradation of formaldehyde at a low concentration [12]. They demonstrated that ultrasound treatment improved the kinetics, producing a final abatement of formaldehyde and breaking the bottleneck of photocatalysis. Santis et al. prepared 2D-SnSe_2_ nanoflakes by using solvent-assisted sonication, and then embedded them in ordered mesoporous titania thin films [13]. The heterostructures formed between SnSe_2_ and nanocrystalline anatase titania resulted in enhanced photocatalytic activity, which was used to remove fingerprints from the surface of smartphones, tablets, and computers. These articles reported highly efficient photocatalysts for water splitting, wastewater treatment, and anti-fingerprint purposes, which are significant for clean energy development and environmental protection. 

In summary, photoactive nanomaterials, encompassing various applications, have attracted enormous research interest. This Special Issue mainly covers the applications of photoactive nanomaterials in photoluminescence, thermochromic smart windows, photocatalytic water splitting, photocatalytic degradation, and functional coatings. We hope that the contributions to this Special Issue enable readers to gain more insight into the recent advances in this area, and also provide helpful guidance for the future development of photoactive nanomaterials.
